# Primary Amyloidosis of Celiac/Para-Pancreatic Lymph Nodes Diagnosed by Endosonography-Guided Fine Needle Aspiration: A Case Report

**DOI:** 10.1177/2324709615607916

**Published:** 2015-09-24

**Authors:** Nuralhuda Akbar, Aahd Kubbara, Ali Nawras

**Affiliations:** 1 University of Toledo Medical Center, Toledo, OH, USA

**Keywords:** primary amyloidosis, para-pancreatic lymph nodes, celiac lymph nodes, fine needle aspiration, endoscopic ultrasound, MGUS, EUS, Congo red

## Abstract

*Introduction*. Primary amyloidosis is a disorder resulting from the deposition of fibrillary protein in extracellular tissue. Diagnosis of primary amyloidosis in the celiac/para-pancreatic lymph nodes via endoscopic ultrasound-guided fine needle aspiration has not been reported in the literature. In this article, we report our first observation. Our patient is a 64-year-old Caucasian man who was referred to our institution from an outlying hospital for recurrent abdominal pain. Radiological imaging revealed an enlarged abdominal lymph node that was already biopsied under computed tomography needle guidance but diagnosis was not achieved on pathological examination. At our institution, endoscopic ultrasound-guided fine needle aspiration showed enlarged para-celiac/pancreatic lymph nodes. Endosonography-guided fine needle aspiration revealed the diagnosis of primary amyloidosis. The patient tolerated the procedure well with follow-up as an outpatient. *Conclusions*. Lymph node involvement in amyloidosis is not uncommon. However, the involvement of the pancreatic/celiac lymph nodes by amyloidosis is obscure in this case. This case shows a rare presentation of amyloidosis diagnosed for the first time by the technique of endosonography-guided fine needle aspiration. In the future, this might serve as an establishment to standardize diagnosing abdominal lymph node amyloidosis, once suspected, by endosonography-guided fine needle aspiration.

## Introduction

Amyloidosis is a disorder resulting from the deposition of fibrillary protein in extracellular tissue. Amyloidosis can be classified into primary, secondary, familial, and senile types. The primary type either manifests without underlying pathology or with plasma cell dyscrasia such as monoclonal gammopathy of undetermined significance (MGUS) as our patient had.

Amyloidosis involvement of lymph nodes results in enlargement. Lymph node involvement in amyloidosis is not uncommon; however, the peculiar involvement of para-pancreatic/celiac lymph nodes in this case is unique. When amyloidosis affects the gastrointestinal tract, the proximal small bowels are the most common parts to be involved. Gastrointestinal involvement can be found in up to 98% of systemic amyloidosis cases.^1^ However, it is uncommon in localized gastrointestinal amyloidosis. Gastrointestinal amyloidosis can present with abdominal pain, malabsorption, pseudo-obstruction, bleeding, perforation, hepatomegaly, diarrhea, and jaundice.^2^

We present a rare case of primary amyloidosis diagnosed using endoscopic ultrasound (EUS) guided fine needle aspiration (FNA) of para-pancreatic/celiac lymph nodes. This is the first reported case of primary amyloidosis diagnosed by EUS-FNA involving the para-celiac/pancreatic lymph nodes.

## Case Presentation

**Figure 1.**
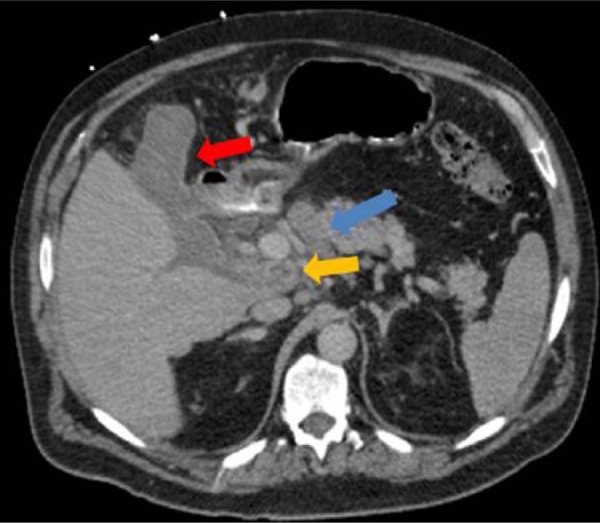
CT scan of the abdomen on presentation to our institution. Description: CT scan at the level of porta hepatis and pancreas showing high-density fluid collection/hemorrhage (red arrow) from the patient’s previous complicated biopsy. There is a mixed attenuated mass between the liver and pancreas (yellow arrow) and a well-defined lesion at the junction between the neck and body of pancreas which represents lymph node (blue arrow).

**Figure 2.**
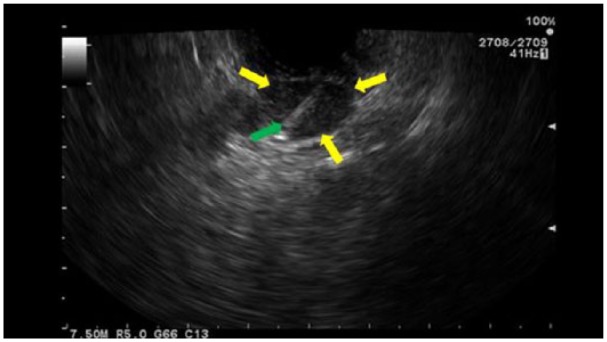
Endosonographic view of the enlarged celiac lymph node. Description: Fine needle aspiration of celiac lymph node. Green arrow is the needle, yellow arrows is the lymph node.

**Figure 3.**
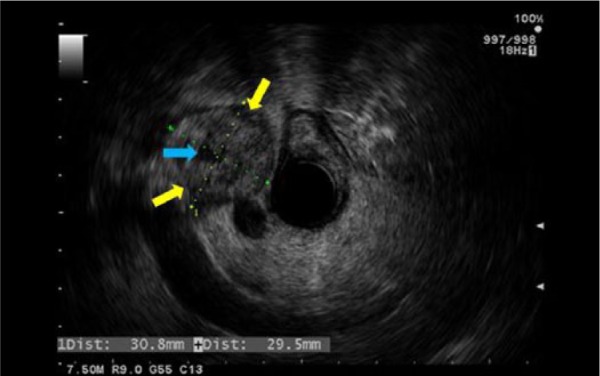
Endosonographic view of the enlarged para-pancreatic lymph node. Description: Endoscopic ultrasound demonstrates a well-defined para-pancreatic head hypoechoic lymph node (LN) measuring 30.8 ′ 29.5 mm (yellow arrow). This LN shows small central anechoic area suggestive of necrosis (blue arrow).

We present a 64-year-old Caucasian gentleman with history of hypertension, hyperlipidemia, gout, and asymptomatic MGUS diagnosed based on serum protein electrophoresis and bone marrow biopsy. He presented initially to an outlying facility with severe right upper quadrant abdominal pain that was colicky in nature. There was associated fatigue, nausea, and vomiting. He denied weight loss or change in bowel habits. His symptoms were of 1 week duration. Abdominal computed tomography (CT) at that time showed 59 mm ′ 39 mm para-pancreatic lymph nodes. CT-guided percutaneous biopsy of the mass was performed to rule out malignancy, which was negative. Diagnosis of amyloidosis was unsuccessful likely due to traumatic tissue sampling. One week later, he presented to our emergency department with recurrent abdominal pain of the same character. CT of the abdomen and pelvis during this visit showed diminished attenuation in a mass between the pancreatic head, body, and liver, consistent with interval hemorrhage within the mass due to the previous biopsy (Figure 1). Pertinent positive labs revealed elevated white blood cell count of 14.1 ′ 10^3^/mL (normal 4-11 ′ 10^3^), alkaline phosphatase level of 288 IU/L (normal 44 to 147 IU/L), and aspartate aminotransferase 58 IU/L (normal 14-20 IU/L). The remaining of the laboratory workup came back normal, including the serum creatinine and urea levels. The patient underwent EUS that revealed multiple celiac and para-pancreatic lymph nodes. The largest celiac lymph node measured 20.0 mm ′ 12.0 mm, and the para-pancreatic lymph node measured 55.0 mm ′ 54.0 mm (Figures 2 and 3). FNA of both lymph nodes with a 25-G needle revealed amorphous material with bland spindle cells on smear slides. Similar amorphous material was present on cell block. Congo red–positive material diagnostic of amyloid was observed as globular deposits admixed with small lymphocytes and plasma cells. Sulfate alcian blue stains were used to confirm the diagnosis of primary amyloidosis. Liquid chromatography tandem mass spectrometry detected a peptide profile consistent with amyloid light-chain (AL) (kappa)-type amyloid deposition. Bone marrow examination showed increased plasma cells consistent with the patient’s history of MGUS. There was also focal increase in amorphous eosinophilic material, which was highly suspicious for amyloidosis. The plasma cell population was 7%, ruling out multiple myeloma. The patient tolerated the procedure well. He was eventually discharged to an inpatient rehabilitation center and carried a slow but successful recovery. He followed-up with our hematology outpatient clinic and did fairly well afterwards with resolution of abdominal pain.

## Discussion

This case presents amyloidosis involving celiac/para-pancreatic lymph nodes diagnosed by EUS-FNA. Amyloidosis refers to a spectrum of diseases characterized by extracellular deposits of protein in tissue and organs in an abnormal fibrillary form resistant to degradation. Amyloidosis can be systemic (80% to 90%) or localized (10% to 20%), acquired or inherited. Systemic amyloidosis often causes death within several years of diagnosis compared with localized amyloidosis, which has a better prognosis. Amyloidosis is classified based on clinical features into (*a*) primary AL amyloidosis, which may be associated with plasma cell disorders including MGUS as in our patient; (*b*) secondary AA amyloidosis, which occurs in the setting of chronic inflammation or infection such as rheumatoid arthritis and osteomyelitis; (*c*) familial AF amyloidosis, which is most commonly due to mutations in the transthyretin, a transport protein; and (*d*) senile amyloidosis.^3^ Amyloidosis can affect many organs, with the most common ones being kidneys, heart, nerves, and gastrointestinal tract. However, the leading causes of death in amyloidosis are cardiac and renal failure.^4^ When amyloidosis affects the gastrointestinal tract, the proximal small bowels are the most common parts to be involved. Gastrointestinal involvement can be found in up to 98% of systemic amyloidosis cases.^1^ However, it is uncommon in localized gastrointestinal amyloidosis. Gastrointestinal amyloidosis can present with abdominal pain as in our patient, who presented with sharp severe colicky abdominal pain due to compressive effect on adjacent organs. It can also present with malabsorption, pseudo-obstruction, bleeding, perforation, hepatomegaly, diarrhea, and jaundice.^2^

Up to 37% of systemic amyloidosis cases have involvement affecting the mediastinal, hilar, and para-aortic lymph nodes.^5^

The diagnosis of amyloidosis involving lymph nodes can be challenging as imaging studies alone are nonspecific. EUS-FNA is useful as it allows cytological examination from the lymph nodes. In all cases, malignancy needs to be ruled out. On accessing the FNA sample, amorphous eosinophilic material must be distinguished from similar material such as non-amyloidic К light chain protein, collagen, and fibrin.

Amyloidosis is associated with some malignancies such as renal cell carcinoma, medullary carcinoma of the thyroid, multiple myeloma, cervical carcinoma, basal cell carcinoma, lymphoma, nasopharyngeal carcinoma, thymoma, Hodgkin’s disease, and plasmacytoma.^3^ In order to diagnose amyloidosis, a tissue biopsy is mandatory. The pathological examination needs special stains, with Congo red in combination with polarized microscopies being the most common method. The characteristic finding is apple-green birefringence appearance. Following that, the subtype of amyloidosis needs to be identified via determining the culprit protein. Several methods exist to identify the protein such as mass spectrometry (as used in our case), immunohistochemistry, immunoelectron microscopy, among others.^6^

MGUS is a disease characterized by a plasma cell proliferative disorder characterized by a plasma cell level of less than 10% in the bone marrow, a monoclonal paraprotein band less than 30 g/L, no end organ damage (CRAB—hyper-calcemia, renal insufficiency, anemia, and bone lesions), and no evidence of another B-cell proliferative disorder. It is rarely complicated by amyloidosis. MGUS is found in more than 1% of persons who are 50 years or older.^7^ When MGUS presents in a patient with biopsy-proven amyloidosis, the AL type should be strongly suspected. MGUS is a well-documented yet rare association of AL amyloidosis.

## Conclusion

MGUS is rarely complicated by amyloidosis. On the other hand, amyloidosis rarely presents in celiac/para-pancreatic lymph nodes. Amyloidosis should be included in the differential diagnosis of abdominal mass/lymphadenopathy especially if there is an association with MGUS. Pathological examination is essential; however, cytology by EUS-FNA can be satisfactory. This case represents a possible establishment for standardizing EUS-FNA in diagnosing intraabdominal lymph node amyloidosis on top of existing CT-guided FNA given the accuracy for sampling the tissue and less complications such as hemorrhage.

## Patient’s Perspective

The patient’s daughter had to say the following:

Originally we went to hospital ER in May 2014 with severe abdominal pain and unrelenting nausea for 1 year. He had stomach pain and severe fatigue, both waxing and waning. For a while symptoms would disappear only to recur later. He also had severe nosebleed. He bled after his GI biopsy as well. Very threatened to have EUS test from Dr Nawras, which showed to have amyloidosis. He tolerated the treatment well. I also noticed skin color change at the wound site significantly over the last year.
